# The Acute Physiological Effects of Multiple Muscle Stimulation

**DOI:** 10.3390/muscles5010010

**Published:** 2026-01-30

**Authors:** Rishabh Rege, Kristin M. Mendez, Riya Patel, Sydnie Keane, Kevin K. McCully

**Affiliations:** 1 Department of Kinesiology, University of Georgia, Athens, GA 30602, USA; rrege@augusta.edu (R.R.); kristin.mendez@uga.edu (K.M.M.); riyapatel1115@gmail.com (R.P.); sskeane@email.sc.edu (S.K.); 2 InfraredRx, Inc., Athens, GA 30602, USA

**Keywords:** neuromuscular electrical stimulation (NMES), muscle metabolism, near-infrared spectroscopy (NIRS), blood flow, mobility limitations

## Abstract

Neuromuscular electrical stimulation (NMES) has been shown to provide health benefits similar to those of exercise. The aim of this study was to quantify the acute physiological effects of multiple muscle stimulation on the whole body and individual muscles. Nine healthy young adults were tested. NMES of eight muscle groups was performed with NMES stimulators. The vastus lateralis, biceps femoris, medial gastrocnemius, and tibialis anterior muscles of both legs were stimulated for ten minutes with twitch stimulations at the highest comfortable stimulation current. Whole-body metabolism was measured using a metabolic cart. A finger pulse oximeter and a tri-axial accelerometer were used to measure heart rate and muscle fatigue, respectively. Muscle metabolism (mVO_2_) was measured using near-infrared spectroscopy (NIRS) during short periods of ischemia. Femoral artery blood flow was measured using Doppler ultrasound. Whole-body VO_2_ and heart rate increased moderately by 36% and 22%, respectively, after 10 min of NMES. NMES increased mVO_2_ by 12-fold higher than resting on average, with the gastrocnemius having the smallest increase and the vastus lateralis having the greatest increase. Peak diastolic blood flow velocity was significantly reduced by 50% after 10 min of NMES. Simultaneous lower-body NMES moderately improved whole-body metabolism, muscle metabolism, and blood flow, increasing our understanding of the beneficial effects of NMES.

## 1. Introduction

Regular exercise is important for maintaining and improving individual health and lowering mortality [1,2,3,4]. Regular exercise is associated with a variety of systemic benefits, including improvements to exercise capacity and overall functional ability [5]. Exercise has also been shown to improve a variety of vascular factors, such as improved endothelial function and reduced arterial blood pressure [6]. Regular exercise is recommended to manage and improve medical conditions like blood sugar and metabolic health [7]. Furthermore, the acute increase in blood flow (sheer stress) on endothelial cells following exercise has proinflammatory effects and reduces the likelihood of atherosclerotic plaque development [8]. Conversely, physical inactivity is associated with systemic disorders such as endothelial dysfunction [9,10].

The ability to exercise may be impaired due to conditions such as peripheral artery disease, multiple sclerosis, muscular dystrophy, or cerebral palsy [11,12]. These conditions can produce increased fatigue, reduced muscle activation, and reduced muscle strength [13]. Reduced ability to exercise then leads to reduced exercise-mediated health benefits. Neuromuscular electrical stimulation (NMES) produces muscle contractions that can provide some of the same physiological responses as exercise [14]. NMES electrode pads are placed directly onto the skin overlying the desired muscle, and allow for direct stimulation of the muscle at a desired frequency and current levels. Thus, NMES has the potential to provide exercise-related health benefits to people with exercise limitations.

Previous studies have used NMES training to produce general health benefits [2,15,16,17]. NMES has also been combined with voluntary exercise to augment training adaptations in people with physical limitations to exercise [18]. One limitation of previous studies using NMES has been a lack of quantification of the physiological stimulus NMES produces. In addition, previous research on the benefits of NMES has stimulated one or two muscle groups. Simultaneous stimulation of multiple muscles may provide substantially greater physiological benefits similar to exercise that involves multiple muscle groups. A better understanding of the physiological impact of multiple-muscle NMES is needed to guide future clinical applications. 

The purpose of this study was to evaluate whole-body and muscle-specific responses to the NMES of eight muscle groups in the legs. We hypothesized that multiple-muscle NMES would produce an 8–12-fold increase in the mVO_2_ of the muscles stimulated based on previous studies and proportionally greater whole-body VO_2_, heart rate, and arterial blood flow, given the simultaneous activation of eight muscle groups.

## 2. Results

Thirteen participants were initially enrolled; however, two subjects discontinued due to discomfort with NMES (15.4%), and two subjects did not complete all three testing sessions (15.4%), yielding a final sample of nine participants (completion rate: 69.2%). The demographics of the subjects who completed the experiment are presented in Table 1 below. An excel file of results are included as Supplementary Material.

**Figure 1:**
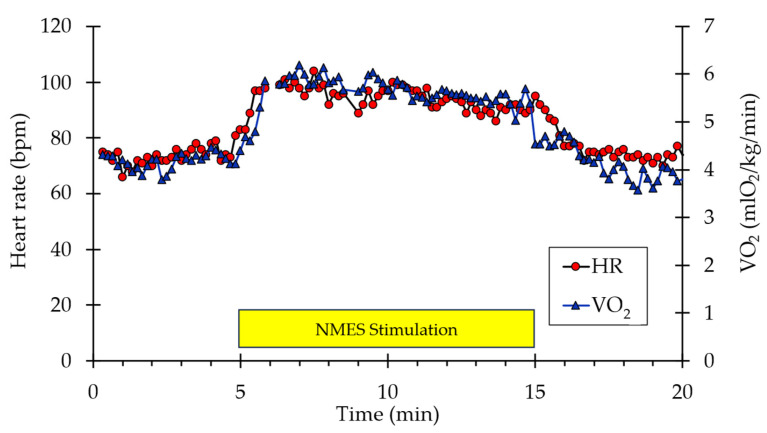
Example of individual whole-body VO_2_ and heart rate data during the experimental protocol. The stimulation period is indicated by the yellow rectangle.

**Figure 2:**
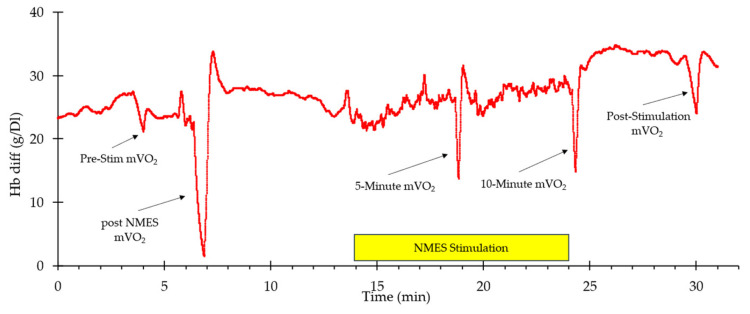
An example of oxygen levels in the vastus lateralis during the experimental protocol. The stimulation period is indicated by the yellow rectangle. The times where mVO_2_ measurements were made are indicated with arrows.

A representative example of whole-body VO_2_ and heart rate responses to NMES is shown in Figure 1. A visible increase in both values can be observed from the start of stimulation to the end (indicated by the box labeled “NMES Stimulation”) before returning to baseline values post-stimulation. Representative muscle oxygen levels from the vastus lateralis are presented in Figure 2. Short duration arterial occlusions were performed at 5 and 10 min of NMES to measure mVO_2_.

**Figure 3:**
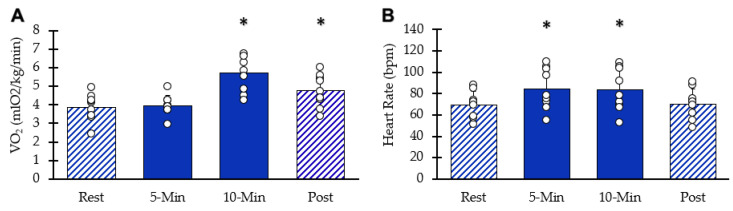
Whole body responses to NMES. (**A**) VO_2_ at each interval. (**B**) Heart rate at each interval. Individual data from participants are represented by the circles. Bars are means with SD. * Indicates significantly different from rest (*p* < 0.05).

Whole-body VO_2_ values at each interval are shown in Figure 3A. After ten minutes of stimulation, VO_2_ increased significantly (95% CI: 4.96–6.44, *p* = 0.00003). Five minutes post-NMES, whole-body VO_2_ remained elevated above resting values (95% CI: 4.08–5.43, *p* = 0.0009). HR values are shown in Figure 3B. Resting heart rate significantly increased with NMES (95% CI: 70–100, *p* = 0.001) at five minutes and at 10 min(95% CI: 68–99, *p* = 0.002). Five minutes post-NMES, heart rate values were not different from baseline values (95% CI: 59.5–81.1, *p* = 0.181). Muscle acceleration values were 81% and 76% of their respective peak values at 5 and 10 min, respectively (*p* < 0.05 for both).

**Figure 4:**
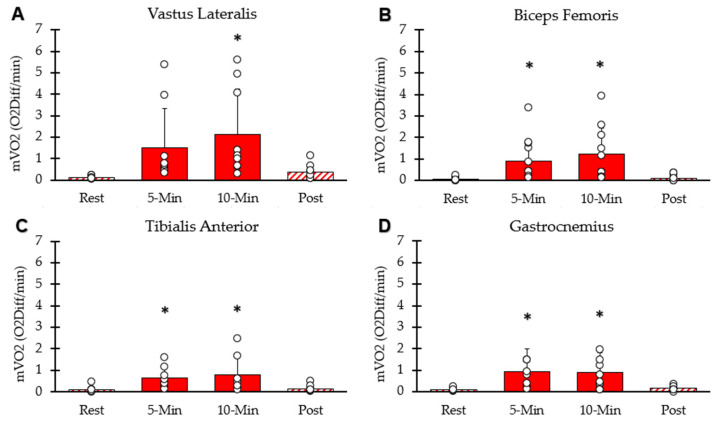
Average mVO_2_ at each interval. (**A**) Vastus lateralis, (**B**) hamstring, (**C**) tibialis anterior, (**D**) gastrocnemius. Individual data from participants are represented by open circles. Bars are means with SD for error bars. * Significantly different from rest (*p* < 0.05).

mVO_2_ of each interval for the four lower-body muscle groups is shown in Figure 4. mVO_2_ values for the four muscle groups were significantly higher than rest at the five-minute (*p* < 0.001) and ten-minute (*p* = 0.002) time points. mVO_2_ calculated as METs was 12.87 ± 7.27 at 5 min, 14.32 ± 7.50 at 10 min, and 2.11 ± 0.85 five minutes post-NMES. The average mVO_2_ from the four muscles at the 10 min time point correlated with whole-body VO_2_ at the 10 min time point (y = 0.82x − 3.4; R^2^ = 0.67, *p* = 0.02).

**Figure 5:**
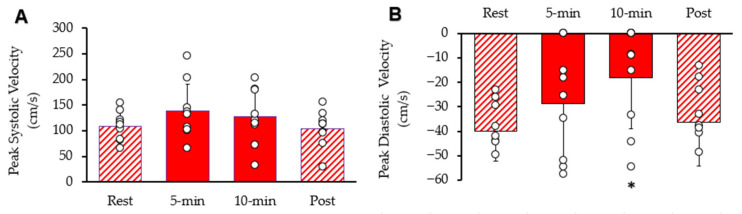
Femoral artery blood velocity during the protocol. (**A**) Peak systolic blood flow velocity and (**B**) peak diastolic blood flow velocity. Individual data from participants are represented by the open circles. Bars are means with SD for error bars. * Significantly different from rest (*p* < 0.05).

Systolic and diastolic blood flow velocity peaks within the common femoral artery are shown in Figure 5. Resting peak systolic blood flow velocity was 1 increased after 5 min 95% CI: 94.94–180.72, *p* = 0.045). After 10 min, peak systolic blood flow velocity was not different from rest (95% CI: 85.88–169.90, *p* = 0.197). It was als not different from rest (95% CI: 76.12–131.89, *p* = 0.389). Resting diastolic blood flow velocity was significantly reduced after ten minutes of stimulation (95% CI: −34.14–−2.33, *p* = 0.011), but not reduced after 5 min stimulation (95% CI: −45.83–−11.31, *p* = 0.097) or post-stimulation (95% CI: −50.11–−22.26, *p* = 0.311). The diameter of the femoral artery was 0.58 cm at rest, 0.59 cm (95% CI: 0.50–0.68, *p* = 0.234) after 5 min of stimulation, 0.60 cm (95% CI: 0.52–0.68, *p* = 0.087) after 10 min of stimulation, and 0.59 cm (95% CI: 0.52–0.66, *p* = 0.279) post-stimulation. Thus, there was no significant change in the diameter of the femoral artery observed during the procedure.

## 3. Discussion

This study found that NMES of multiple lower-body muscle groups produced an increased whole-body response equivalent to 1.5 METs (metabolic equivalents). These results can be compared to whole body MET levels for sitting and standing (1.2–1.3 METs), slow walking (3.0 METs), brisk walking (5.4 METs), and running (8.2 METs) [19]. The increase in MET values seen in this study was much less than the guidelines for able-bodied adults, which is 150 min to 300 min a week of moderate-intensity physical activity (3.0–5.9 METs) [19]. However, people with mobility limitations are less likely to achieve these guidelines. Even so, they may achieve physiological benefits at lower activity levels [11,20]. For example, performing 80 NMES muscle contractions per week produced a 37% increase in mVO_2_ max in people with motor complete spinal cord injury [21]. Similarly, the significant increase in heart rate during stimulation by an average of 14 bpm after 10 min seen in this study could result in mild cardiovascular benefits for these populations. In this study the NMES protocol significantly fatigued the quadriceps, with twitch acceleration reduced 24% after 10 min of stimulation. This result suggests that NMES was sufficient to provide a metabolic stimulus that could lead to muscular adaptations over multiple sessions [22].

NMES of the lower body produced large increases in muscle metabolism (mVO_2_). mVO_2_ increased to 7–17 METS in the different muscle groups during stimulation. This was comparable to previous studies that reported an 8–12-fold increase in muscle metabolism with single-muscle NMES [23]. Differences in average mVO_2_ between muscles can be attributed to inherent differences in size. A previous NMES study, which stimulated four muscles in one leg, found up to 6-fold increases in the muscle metabolic rate of oxygen [24]. The muscle MET values collected in this study were also higher than the muscle MET values of 3.2, 4.5, and 5.8 measured in the vastus lateralis muscles during treadmill walking at 3.3, 5, and 6.7 km/h [25]. The ability of NMES to produce significant increases in mVO_2_ supports its value in improving the health of these muscles in mobility-impaired populations. It is also important to note that the significant increases in mVO_2_ during stimulation did not produce equivalent fold increases in whole-body VO_2_. This is likely due to the localization of the NMES to specific muscles, as opposed to exercises such weight-training or running which can recruit more than eight muscle groups. It would be of interest to lengthen the protocol in future studies to determine whether long durations of multiple-muscle NMES can cause more significant increases in the whole-body response.

The NMES used in this study produced modest changes in blood flow in the common femoral artery. Previous studies have explored the relationship between increased likelihood of disease and cardiovascular degradation with the stagnant and retrograde blood flow in individuals with impaired mobility [4,8]. By reducing retrograde blood flow by approximately 50% after 10 min of NMES, it is possible that NMES could reduce the production of inflammatory cytokines and reduce the risk of infection within the arteries. This can be attributed to improved vascular function, as corroborated by one study, which found that NMES improved flow-mediated dilation (a marker of endothelial function) and increased lower-limb blood flow in patients with PAD [26]. Obtaining greater changes in arterial flow with NMES may require adding additional interventions, such as localized warming [27] or passive limb movements [28].

A key aspect of this study was the participants’ tolerance to electrical stimulation. In our experiment, we included subjects with low tolerance for NMES. This resulted in low activation levels and lower-than-expected mVO_2_ and blood flow values. A prior study used NMES on one muscle group (tibialis anterior) and obtained much higher muscle blood flow values [23]. Despite the small sample size, we did observe a significant correlation between whole-body VO_2_ values and the average muscle mVO_2_ values at the 10 min time point. This suggests that tolerating higher stimulation currents is important in determining the whole-body VO_2_. Many of the subjects in our study were not used to electrical stimulation, and it is possible that habituation to NMES would result in tolerating higher stimulation currents and thus produce greater physiological responses to NMES. Populations of people with low activity levels may have higher sensitivity to NMES, or lower sensitivity. People with motor complete spinal cord injury have shown a tolerance for high current levels, and NMES using twitch contractions similar to the ones used in this study has been successfully applied for 4 months, with stimulation durations of up to 70 min per session. In this population, meaningful increases in muscle endurance, mVO2max (137% on average), and vascular health have been reported [29,30]. Thus, certain patient populations may not only be able to tolerate higher stimulation levels but would also receive much greater increases in whole-body and muscle-specific physiological adaptations. Additional studies on the use of multiple-muscle NMES on specific patient populations would be of great value.

The stimulation protocol used in this study was twitch electrical stimulation at 6 Hz. The advantages of this stimulation pattern are that it produces relatively high muscle energetic responses with low force development. Furthermore, 6 Hz provides the highest stimulation frequency that does not result in summation (tetanus). Twitch contractions are too brief to generate high force levels or large limb movements [31]. Lower force levels and small limb displacements reduce the likelihood of muscle, bone, or connective tissue injury. In addition, twitch contractions are better tolerated than tetanic contractions, allowing for more muscle activation in people who have a lower tolerance for NMES. Twitch contractions can also have higher energetic costs due to a greater proportion of the contraction time being in the concentric (shortening) phase of contraction [32]. This makes meeting the energetic goals of muscle exercise more likely with twitch contractions than isometric tetanic contractions. A recent previous study concluded that twitch contractions provided an optimal stimulus for people with spinal cord injuries [33]. A limitation to the use of twitch contractions is the lack of a muscle hypertrophy stimulus, due to the lower force levels. In the future, optimal training protocols using NMES may include a combination of twitch stimulation to produce an endurance stimulus [29] and tetanic contractions to produce a hypertrophy stimulus [21].

There were several limitations to this study. This study had a small sample size of healthy young adults. The benefits as well as challenges to performing NMES in multiple muscle groups needs to be extended to larger studies, and to studies of mobility-limited participants. Future studies could also extend the findings of this study to evaluating potential changes in metabolic health (blood sugar and insulin levels) as well as in immune and inflammatory status, for example, evaluating whether relatively small amounts of exercise such as walking for 10 min can lower post-prandial blood sugar levels in people with impaired glucose regulation [7]. This study also recruited participants who were naïve to NMES. To optimize the benefits of NMES, experience with NMES should allow subjects to tolerate higher levels of electrical stimulation, especially as using NMES on eight different muscle groups at the same time is a more intense overall stimulus that NMES of one or even two muscle groups. Overall, these limitations suggest that this study can serve as a preliminary guide to future studies examining the benefits of multiple muscle stimulation.

## 4. Materials and Methods

### 4.1. Participant

The participants were undergraduate students from the University of Georgia who were free of significant disease and any injury that could have affected their legs. The study was approved by the Institutional Review Board for the protection of human participants at the University of Georgia, and all participants provided informed consent.

### 4.2. Experimental Design

The study consisted of a single group with three testing sessions per participant. Each session consisted of 5 min of rest, 10 min of electrical stimulation of the eight muscle groups, and 5 min of recovery. Participants were advised in each session to remain quiet and breathe normally throughout the procedure. They were also asked to remain awake during the procedure. Testing occurred in a closed room.

The three sessions included the following: whole-body metabolic measurements, vascular measurements, and muscle metabolic measurements performed in varying order on the participants’ right legs. These testing sessions were completed within two weeks, with at least 24 h between each session. Participants avoided strenuous exercise for 24 h before each testing session. 

Neuromuscular electrical stimulation was performed using four-channel Theratouch EX4 and 4.7 devices (Richmar, Clayton, MO, USA). Stimulation was performed by attaching two 90 × 50 mm rectangular electrode pads to each of the eight muscle groups in the legs. These muscle groups include the lateralis femoris, biceps femoris, gastrocnemius (medial and lateral heads), and tibialis anterior of both the left and right legs. In some subjects, smaller 76 mm diameter circular pads were placed on the appropriate muscle. Both stimulators were programmed to produce 200 µs biphasic pulses at 6 Hz. The stimulation rate of 6 Hz was chosen as the highest stimulation rate that produces individual twitch contractions (without summation). The current levels were set for each muscle at the highest level the subject felt comfortable with, typically at a pain level of 6 on a scale of 1–10. Stimulation frequency and current remained constant across all the sessions.

Whole-body metabolism, heart rate, and muscle endurance were collected in one session. Whole-body VO_2_ was measured using indirect calorimetry with a metabolic cart (Parvo with VO_2_ Master software, version 0.49.0 Salt Lake City, UT, USA). The machine was calibrated to atmospheric values before the participant’s arrival. The participants were positioned supine on a bed and allowed to rest for 10 min before data collection started. A canopy system (Parvo TrueOne 2400) was placed over the participant’s head and tucked around the participant’s body. The cart continuously measured the metabolic rate of the participant throughout the procedure, using the manufacturer’s recommended procedures. The first 5 min were used to calibrate the VO_2_ system. Heart rate was measured by placing a finger pulse oximeter (Santa Medical, Tustin, CA, USA) on the participant’s index finger. Muscle contraction strength was collected by placing a tri-axial accelerometer (Mbientlab, San Francisco, CA, USA) on the rectus femoris of the right leg.

Skeletal muscle metabolism (mVO_2_) was measured in one testing session as the rate of oxygen consumption during a short period of arterial occlusion [34,35]. Previous studies have demonstrated the reliability and reproducibility of the NIRS method of evaluating muscle metabolism [34,36,37,38]. Two NIRS devices (Portamon, Artinis Ltd., Elst, The Netherlands) were attached to the quadriceps and hamstrings between the stimulation pads and secured by athletic tape. Two NIRS devices (TrainRed Plus, TrainRed, Elst, The Netherlands) were attached to the gastrocnemius and tibialis anterior muscles in a similar fashion. Tissue oxygen index (TSI) values were collected at 10 Hz throughout the experimental protocol. Arterial occlusion was produced using an occlusion cuff (V24, Delfi, Vancouver, BC, Canada) on the inguinal fold of the right side. The cuff was connected to a rapid cuff inflator device (E20, Hokanson, Milwaukee, WI, USA) and a 30-gallon air compressor tank, set to approximately 250 mmHg. A pillow was placed under the participant’s knee joint for comfort. mVO_2_ was measured at rest, at the 5 min and 10 min time points of NMES, and 5 min after the NMES was turned off. Arterial occlusion was produced with 250 mm Hg of pressure for 10 s, and the slope of the TSI signal was measured as the index of mVO_2_.

Arterial blood flow was measured in one testing session. The participant laid supine on the bed with placement of the electrode pads. A handheld ultrasound (Clarius, Vancouver, BC, Canada) was placed along the common femoral artery of the right leg. Femoral artery diameter and blood flow velocity were measured via B-mode ultrasound and pulse wave Doppler. Representative waveforms were collected at rest, after 5 and 10 min of NMES, and 5 min post-NMES. Image J software (version 1.54k) was used to measure arterial diameter and peak systolic and diastolic blood flow velocity. Stimulation had to be turned off momentarily for blood flow to be measured, and this time was recorded. The blood flow velocity was converted into blood flow rate using the diameter measurements of the artery.

### 4.3. Analysis

Descriptive statistics used means and standard deviations. Statistical comparisons were made comparing values during NMES and recovery to resting values. Preliminary power calculations assumed a difference between groups of 15% and a standard deviation of the sample of 15%. Based on this, an adequate power (b = 0.8) with a level of significance (*p* = 0.05) could be achieved with a sample size of 10. Statistical significance was assumed with *p* values < 0.05.

## 5. Conclusions

This study demonstrated that large changes in muscle metabolism can be produced in multiple muscles in the lower leg with eight-muscle NMES. Despite this, the relative changes in whole-body metabolism and leg arterial blood flow were modest, being equivalent to the metabolic changes during very light exercise. Higher levels of NMES were possible but not performed due to subject discomfort. Higher stimulation currents may be achieved by using habituation procedures or in populations with sensory nerve loss. Future studies may focus on the health benefits related to changes in muscle metabolism produced by NMES, such as changes in blood glucose levels after meals, in addition to cardiovascular benefits. Because NMES using twitch muscle contractions can be easily performed with relatively inexpensive equipment, more research into the mechanisms underlying potential health benefits is warranted.

## Figures and Tables

**Table 1:** Baseline characteristics of participants who completed the study.

Age, yr	BMI, kg/m^2^	Sex, M/F	Height, cm	Weight, kg
20.2	22.8	Female *N* = 6	160.8	58.8
(1.2)	(2.1)		(6.5)	(3.9)
20.3	22.8	Male *N* = 3	178.0	72.6
(1.5)	(1.0)		(8.0)	(9.3)

Values are expressed as means (SD). M, male; F, female.

## Data Availability

Data are available upon request.

## Supplementary Materials

The following supporting information can be downloaded at: https://www.mdpi.com/article/10.3390/muscles5010010/s1, Data file: MMS Summary Graph.
